# Supplementary material for: Giants in the landscape: status, genetic diversity, habitat suitability and conservation implications for a fragmented Asian elephant (Elephas maximus) population in Cambodia
Source: PeerJ. 2025 Mar 13;13:e18932. doi: 10.7717/peerj.18932 (PMC11910960; doi:10.7717/peerj.18932)
Supplement: Supplemental Information 2 [file peerj-13-18932-s002.docx]

LOCUS PLEL_haplotype_1-BN 152 bp DNA circular MAM 31-DEC-2023

DEFINITION mitochondrial Dloop from Cambodia.

ACCESSION PLEL_haplotype_1-BN

VERSION

KEYWORDS .

SOURCE mitochondrion Elephas maximus (Asiatic elephant)

ORGANISM Elephas maximus

Eukaryota; Metazoa; Chordata; Craniata; Vertebrata; Euteleostomi;

Mammalia; Eutheria; Afrotheria; Proboscidea; Elephantidae; Elephas.

REFERENCE 1 (bases 1 to 152)

AUTHORS Sinovas,P., Smith,C., Keath,S., Chantha,N., Kaden,J., Ith,S. and

Ball,A.

TITLE Status, genetic diversity, habitat suitability and conservation

implications for a fragmented Asian elephant (Elephas maximus)

population in Cambodia

JOURNAL unpublished

REFERENCE 2 (bases 1 to 152)

AUTHORS Ball,A., Kaden,J. and Keath,S.

TITLE Direct Submission

JOURNAL Submitted (14-MAY-2023) RZSS WildGenes, Royal Zoological Society of

Scotland, 134 Corstorphine Road, Edinburgh, EH12 6TS, United

Kingdom

COMMENT Bankit Comment: ALT EMAIL:aball@rzss.org.uk

Bankit Comment: TOTAL # OF SEQS:4

##Assembly-Data-START##

Sequencing Technology :: Sanger dideoxy sequencing

##Assembly-Data-END##

FEATURES Location/Qualifiers

source 1..152

/organism="Elephas maximus"

/organelle="mitochondrion"

/mol_type="genomic DNA"

/bio_material="faecal"

/db_xref="taxon:9783"

/country="Cambodia"

/collection_date="2021"

BASE COUNT 45 a 29 c 25 g 53 t

ORIGIN

1 caagcactgt ttagtcaatg tgttgagtca tattcttgta gattcacagg ttatgtttta

61 gctcatggat attattcacc tacgataaac catagtctta catagcacat taaagctctt

121 gatcgtacat agcgcattac tgagaaatct ct

//

LOCUS PLEL_haplotype_2-AAAK 152 bp DNA circular MAM 31-DEC-2023

DEFINITION mitochondrial Dloop from Cambodia.

ACCESSION PLEL_haplotype_2-AAAK

VERSION

KEYWORDS .

SOURCE mitochondrion Elephas maximus (Asiatic elephant)

ORGANISM Elephas maximus

Eukaryota; Metazoa; Chordata; Craniata; Vertebrata; Euteleostomi;

Mammalia; Eutheria; Afrotheria; Proboscidea; Elephantidae; Elephas.

REFERENCE 1 (bases 1 to 152)

AUTHORS Sinovas,P., Smith,C., Keath,S., Chantha,N., Kaden,J., Ith,S. and

Ball,A.

TITLE Status, genetic diversity, habitat suitability and conservation

implications for a fragmented Asian elephant (Elephas maximus)

population in Cambodia

JOURNAL unpublished

REFERENCE 2 (bases 1 to 152)

AUTHORS Ball,A., Kaden,J. and Keath,S.

TITLE Direct Submission

JOURNAL Submitted (14-MAY-2023) RZSS WildGenes, Royal Zoological Society of

Scotland, 134 Corstorphine Road, Edinburgh, EH12 6TS, United

Kingdom

COMMENT Bankit Comment: ALT EMAIL:aball@rzss.org.uk

Bankit Comment: TOTAL # OF SEQS:4

##Assembly-Data-START##

Sequencing Technology :: Sanger dideoxy sequencing

##Assembly-Data-END##

FEATURES Location/Qualifiers

source 1..152

/organism="Elephas maximus"

/organelle="mitochondrion"

/mol_type="genomic DNA"

/bio_material="faecal"

/db_xref="taxon:9783"

/country="Cambodia"

/collection_date="2021"

BASE COUNT 44 a 29 c 27 g 52 t

ORIGIN

1 caagtactgt ttagtcaatg tgtcaagtca tatttgtgta gatccacagg tcatgttcta

61 gttcatggat attgttcacc tacgataaac catagtctta catagcacat taaagctctt

121 gatcgtgcat agcgcattac tgagaaattt ct

//

LOCUS PLEL_haplotype_3-AC 152 bp DNA circular MAM 31-DEC-2023

DEFINITION mitochondrial Dloop from Cambodia.

ACCESSION PLEL_haplotype_3-AC

VERSION

KEYWORDS .

SOURCE mitochondrion Elephas maximus (Asiatic elephant)

ORGANISM Elephas maximus

Eukaryota; Metazoa; Chordata; Craniata; Vertebrata; Euteleostomi;

Mammalia; Eutheria; Afrotheria; Proboscidea; Elephantidae; Elephas.

REFERENCE 1 (bases 1 to 152)

AUTHORS Sinovas,P., Smith,C., Keath,S., Chantha,N., Kaden,J., Ith,S. and

Ball,A.

TITLE Status, genetic diversity, habitat suitability and conservation

implications for a fragmented Asian elephant (Elephas maximus)

population in Cambodia

JOURNAL unpublished

REFERENCE 2 (bases 1 to 152)

AUTHORS Ball,A., Kaden,J. and Keath,S.

TITLE Direct Submission

JOURNAL Submitted (14-MAY-2023) RZSS WildGenes, Royal Zoological Society of

Scotland, 134 Corstorphine Road, Edinburgh, EH12 6TS, United

Kingdom

COMMENT Bankit Comment: ALT EMAIL:aball@rzss.org.uk

Bankit Comment: TOTAL # OF SEQS:4

##Assembly-Data-START##

Sequencing Technology :: Sanger dideoxy sequencing

##Assembly-Data-END##

FEATURES Location/Qualifiers

source 1..152

/organism="Elephas maximus"

/organelle="mitochondrion"

/mol_type="genomic DNA"

/bio_material="faecal"

/db_xref="taxon:9783"

/country="Cambodia"

/collection_date="2021"

BASE COUNT 45 a 28 c 26 g 53 t

ORIGIN

1 caagtactgt ttagtcaatg tgtcaagtca tatttgtgta gatccacagg ttatgttcta

61 gttcatggat attgttcacc tacgataaac catagtctta catagcacat taaagctctt

121 gatcgtacat agcgcattac tgagaaattt ct

//

LOCUS PLEL_haplotype_4-ADAE 152 bp DNA circular MAM 31-DEC-2023

DEFINITION mitochondrial Dloop from Cambodia.

ACCESSION PLEL_haplotype_4-ADAE

VERSION

KEYWORDS .

SOURCE mitochondrion Elephas maximus (Asiatic elephant)

ORGANISM Elephas maximus

Eukaryota; Metazoa; Chordata; Craniata; Vertebrata; Euteleostomi;

Mammalia; Eutheria; Afrotheria; Proboscidea; Elephantidae; Elephas.

REFERENCE 1 (bases 1 to 152)

AUTHORS Sinovas,P., Smith,C., Keath,S., Chantha,N., Kaden,J., Ith,S. and

Ball,A.

TITLE Status, genetic diversity, habitat suitability and conservation

implications for a fragmented Asian elephant (Elephas maximus)

population in Cambodia

JOURNAL unpublished

REFERENCE 2 (bases 1 to 152)

AUTHORS Ball,A., Kaden,J. and Keath,S.

TITLE Direct Submission

JOURNAL Submitted (14-MAY-2023) RZSS WildGenes, Royal Zoological Society of

Scotland, 134 Corstorphine Road, Edinburgh, EH12 6TS, United

Kingdom

COMMENT Bankit Comment: ALT EMAIL:aball@rzss.org.uk

Bankit Comment: TOTAL # OF SEQS:4

##Assembly-Data-START##

Sequencing Technology :: Sanger dideoxy sequencing

##Assembly-Data-END##

FEATURES Location/Qualifiers

source 1..152

/organism="Elephas maximus"

/organelle="mitochondrion"

/mol_type="genomic DNA"

/bio_material="faecal"

/db_xref="taxon:9783"

/country="Cambodia"

/collection_date="2021"

BASE COUNT 44 a 28 c 27 g 53 t

ORIGIN

1 caagtactgt ttagtcaatg tgtcaagtca tatttgtgta gatccacagg ttatgttcta

61 gttcatggat attgttcacc tacgataaac catagtctta catagcacat taaagctctt

121 gatcgtgcat agcgcattac tgagaaattt ct

//
