# Supplementary figures and images for: Giants in the landscape: status, genetic diversity, habitat suitability and conservation implications for a fragmented Asian elephant (Elephas maximus) population in Cambodia
Source: PeerJ. 2025 Mar 13;13:e18932. doi: 10.7717/peerj.18932 (PMC11910960; doi:10.7717/peerj.18932)

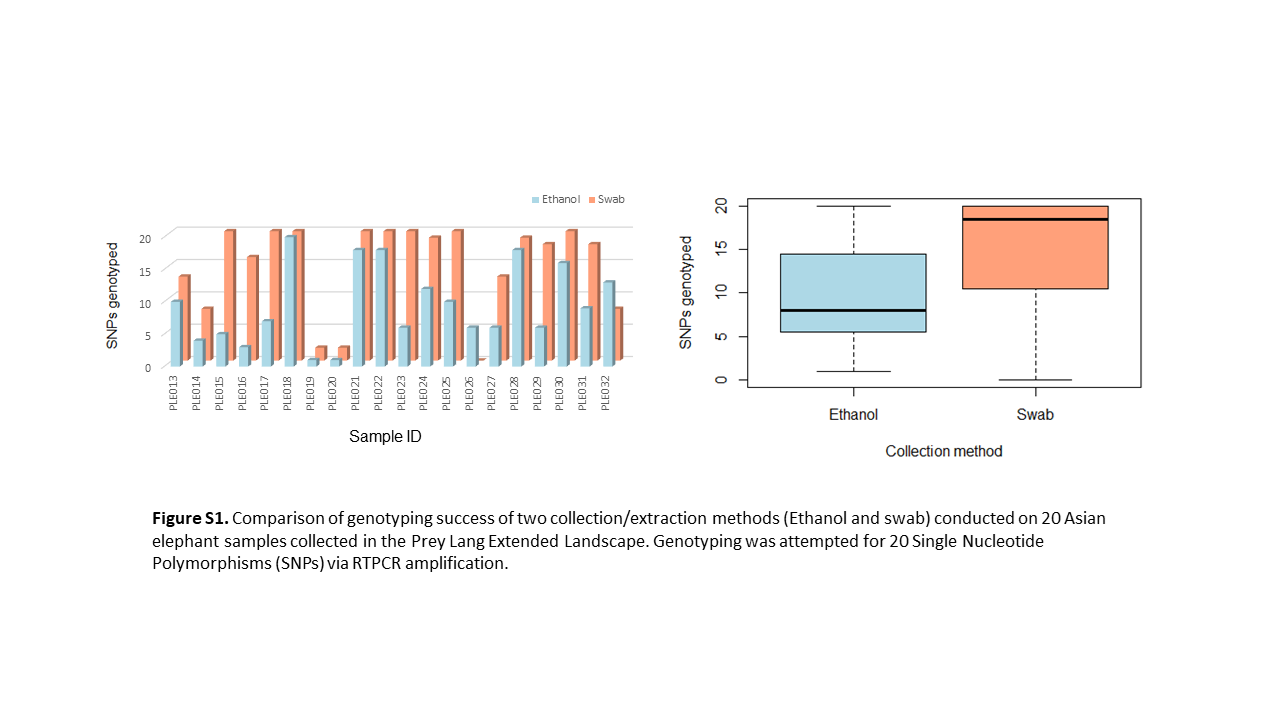

Supplement: Supplemental Information 3 — Genotyping was attempted for 20 Single Nucleotide Polymorphisms (SNPs) via RTPCR amplification. [file peerj-13-18932-s003.png]

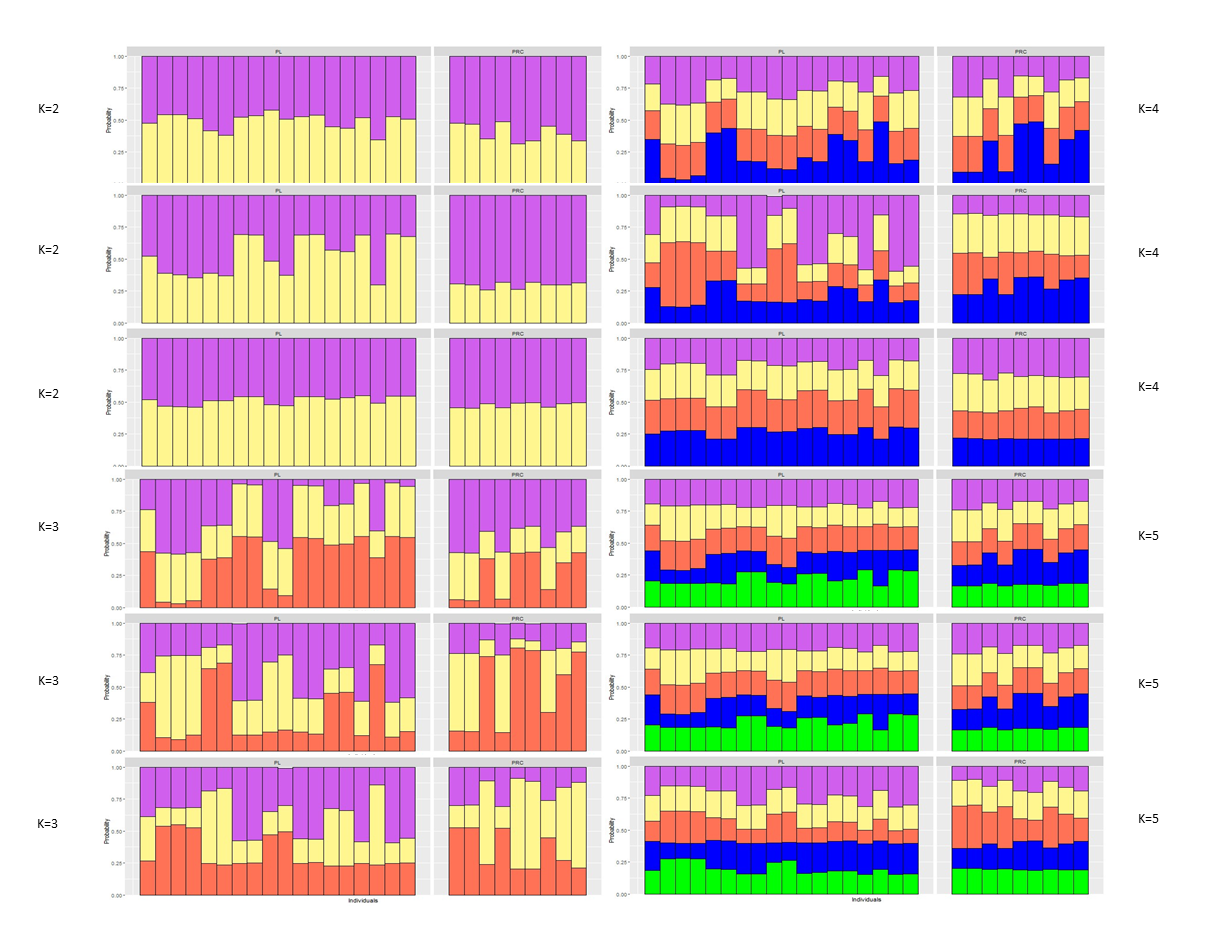

Supplement: Supplemental Information 4 — A priori population sizes for k=2 to k=6 are shown, and each analysis was run in triplicate. The results are consistent between the replicates. Prior to analysis unique genotypes that had been scored at <4 microsatellites were removed (n=8). [file peerj-13-18932-s004.png]
