# Supplementary material for: Giants in the landscape: status, genetic diversity, habitat suitability and conservation implications for a fragmented Asian elephant (Elephas maximus) population in Cambodia
Source: PeerJ. 2025 Mar 13;13:e18932. doi: 10.7717/peerj.18932 (PMC11910960; doi:10.7717/peerj.18932)
Supplement: Supplemental Information 5 [file peerj-13-18932-s005.docx]

**Supplementary Table S1:**

**Details of the KASP assays for the 20 SNPs used for individual identification in this study.**

| Assay ID | FAM Allele | HEX Allele | Sequence |
| --- | --- | --- | --- |
| AEL_SNP002 | C | T | CTAACCTCACCTCCT**[C/T]**TTCCTGTGCTACCTG |
| AEL_SNP004 | A | C | TAGGGTCAGGCTTAG**[A/C]**TCTTCTCCTCTGCCA |
| AEL_SNP005 | A | C | CTCTAGCAGAACTCC**[A/C]**AGGTGTATGAGTAAT |
| AEL_SNP010 | C | T | ATGTATGTACAGCTC**[C/T]**TCACATGGTGCCTGG |
| AEL_SNP011 | C | T | CAGGATCTACAGGCT**[C/T]**CTCCTGGGAGCATCC |
| AEL_SNP013 | A | C | CTATGCTTGAGCTAG**[A/C]**TACTGAGACTGCACC |
| AEL_SNP016 | C | T | TCCCTCCTCCAAGCC**[C/T]**GCCTTTAGGAGTGGG |
| AEL_SNP021 | A | G | GATAGCTTTTCCAGC**[A/G]**CCGGCTTTGGTGTGT |
| AEL_SNP023 | A | T | AAGTGCCAAAGCCTC**[A/T]**AGATCTATTTGGTGC |
| AEL_SNP025 | G | T | AATTGTATTTAATTT**[G/T]**CTGTGGCTCAAGGGC |
| AEL_SNP026 | G | C | AGTAAAAAAGTATTG**[G/C]**GTTTTAATTACTTAT |
| AEL_SNP027 | G | T | GGTAGGCTGAGAGAT**[G/T]**GGGAGAGGCTGGCAC |
| AEL_SNP029 | A | C | TTCCCAAGACATGCC**[A/C]**GGGTAATTGGTCCCA |
| AEL_SNP031 | A | G | TTCAAGTCACTTGGA**[A/G]**AATAAGAGTTTTTTC |
| AEL_SNP033 | A | G | GTCGCAGGGACCAGC**[A/G]**TCTTCCAGTTTGAAT |
| AEL_SNP035 | C | T | CACTCTTCTCTCTTG**[C/T]**TGCCCTGACAGTCTG |
| AEL_SNP036 | G | C | ACCATAACCACATTG**[G/C]**CAGCCAGTCATAAAA |
| AEL_SNP037 | G | C | TAGATCCAGACACTG**[G/C]**GACTCCAGATACATA |
| AEL_SNP039 | C | T | CCAAGTGGAGGCAAG**[C/T]**GGGTTCCCTCAATTT |
| AEL_SNP040 | A | G | GGCAGAGTTCAGGGA**[A/G]**TAGTTGTTGACTTAT |
