# Supplementary material for: Giants in the landscape: status, genetic diversity, habitat suitability and conservation implications for a fragmented Asian elephant (Elephas maximus) population in Cambodia
Source: PeerJ. 2025 Mar 13;13:e18932. doi: 10.7717/peerj.18932 (PMC11910960; doi:10.7717/peerj.18932)
Supplement: Supplemental Information 6 [file peerj-13-18932-s006.docx]

**Supplementary Table S2:**

**Microsatellite, mitochondrial and sexing primers used in this study.**

| **Oligo Name** | **Sequence (5' to 3')** | **5' Dye** | **Reference** |
| --- | --- | --- | --- |
| **Genotyping:** |  |  |  |
| **Microsatellite multiplex 1 (P1):** |  |  |  |
| Emu03_Eleph_F | AGAAGCAAAACCCATGAAGC | NED | Kongrit et al. 2008 |
| Emu03_Eleph_R | TTGAAACTTGCCAGCCTCTT |  |  |
| Emu04_Eleph_F | TGACTCTCCCTCTTCTGCATC | FAM | Kongrit et al. 2008 |
| Emu04_Eleph_R | GGCTGAGAGGGAAAGAAATTG |  |  |
| Emu07_Eleph_F | GAGCAGTGCCTTTCGTGAC | VIC | Kongrit et al. 2008 |
| Emu07_Eleph_R | AGCCTGGGAGGTAAGTAGCA |  |  |
| Emu12_Eleph_F | CAAAGAAGACCCATGTTCC | PET | Kongrit et al. 2008 |
| Emu12_Eleph_R | CTGACTATGGGGGAGACTGC |  |  |
| FH48_Eleph_F | GAGTCTCCATAATCAAGAGCG | FAM | Comstock et al. 2000 |
| FH48_Eleph_R | CCTCCCTGGAATCTGTACAG |  |  |
| **Microsatellite multiplex 2 (P2):** |  |  |  |
| Emu10_Eleph_F | AATCGACTCAGCAGCAACAG | FAM | Kongrit et al. 2008 |
| Emu10_Eleph_R | CCAGTAAATCCATATCACTCGTC |  |  |
| Emu15_Eleph_F | TTCGGGATGTTCTCTTCTGT | NED | Kongrit et al. 2008 |
| Emu15_Eleph_R | GGGGCTTAACTAATAGGCTTCA |  |  |
| Emu17_Eleph_F | CACTCAGAGTTCCAAGAAGCAG | VIC | Kongrit et al. 2008 |
| Emu17_Eleph_R | TGCCAGCCATTTCCTCTC |  |  |
| LafMS03_Eleph_F | CATATGAACATACCGGAAC | PET | Nyakaana and Arctander 1998 |
| LafMS03_Eleph_R | GAAACTCCTCGAGTAGTAGAA |  |  |
| **Mitochondrial:** |  |  |  |
| AEL_dloop_1360_F | GCATCACATTATTTACCCCATGC | N/A | This study |
| AEL_dloop_1564_R | GGTTGATGGTTTCTCGGAGG |  |  |
| CR_F1_AEL_RZSS | TTAAATGCTCGTCCCCATACA | N/A | This study |
| CR_R1_AEL_RZSS | ACGATCAAGAGCTTTAATGTGC |  |  |
| **Sexing:** |  |  |  |
| PLP1_Ele_SEXF | CCCACTTCTGCCATATCTGC | N/A | Ahlering et al. 2011 |
| PLP1_Ele_SEXR | GCAGAGGCTCCAACTCAATC |  |  |
| AMELY2_Ele_SEX_F | CAGGGAGGTTTTACGTTAGGG | N/A | Ahlering et al. 2011 |
| AMELY2_Ele_SEX_R | GCGATTTGAAGCTGAAAAGG |  |  |
| SRY1_Ele_SEX_F | CCAGTGGAAAATGCTTACGG | N/A | Ahlering et al. 2011 |
| SRY1_Ele_SEX_R | GCATTGCCCTTAGTCTCTGC |  |  |
