# Supplementary material for: Giants in the landscape: status, genetic diversity, habitat suitability and conservation implications for a fragmented Asian elephant (Elephas maximus) population in Cambodia
Source: PeerJ. 2025 Mar 13;13:e18932. doi: 10.7717/peerj.18932 (PMC11910960; doi:10.7717/peerj.18932)
Supplement: Supplemental Information 7 — These were selected based on a literature review and guidance from experts consulting on variables likely to impact Asian elephant habitat. An updated forest cover layer was created by reclassifying deforestation events from 2011-2020 as deforested in the 2010 canopy cover dataset. Deforestation hot spots were identified by aggregating forest lost1 in 1 km2 grid events, using the create space-time cube and emerging hot spots tool in ArcGIS Pro 2.8 to evaluate spatio-temporal trends. The analysis was run for historical deforestation (2001 to 2015) and deforestation inclusive of recent events (2001 to 2020). Euclidean distance was applied to recent and historic deforestation hotspots, defined as new, consecutive, intensifying and persistent hot spots, village and river datasets. The deforestation, village and road density variables were used to represent anthropogenic disturbance in the PLEL. Fuzzification transformations were applied to variables for the weighted fuzzy model. Hansen, M. C., P. V. Potapov, R. Moore, M. Hancher, S. A. Turubanova, A. Tyukavina, D. Thau, S. V. Stehman, S. J. Goetz, T. R. Loveland, A. Kommareddy, A. Egorov, L. Chini, C. O. Justice, and J. R. G. Townshend. 2013. “High-Resolution Global Maps of 21st-Century Forest Cover Change.” Science 342 (15 November): 850–53. Data available from: https://glad.umd.edu/dataset/global-2010-tree-cover-30-m. [file peerj-13-18932-s007.docx]

**Table S3:**

**Variables and fuzzification parameters used in habitat suitability modelling.**

These were selected based on a literature review and guidance from experts consulting on variables likely to impact Asian elephant habitat. An updated forest cover layer was created by reclassifying deforestation events from 2011-2020 as deforested in the 2010 canopy cover dataset^[[1]](#footnote-1)^. Deforestation hot spots were identified by aggregating forest lost^1^ in 1km^2^ grid events, using the create space-time cube and emerging hot spots tool in ArcGIS Pro 2.8 to evaluate spatio-temporal trends. The analysis was run for historical deforestation (2001 to 2015) and deforestation inclusive of recent events (2001 to 2020). Euclidean distance was applied to recent and historic deforestation hotspots, defined as new, consecutive, intensifying and persistent hot spots, village and river datasets. The deforestation, village and road density variables were used to represent anthropogenic disturbance in the PLEL. Fuzzification transformations were applied to variables for the weighted fuzzy model.

| **1. Variable** | **2. Habitat Relationship** | **3. Fuzzification** | **4. Variable Weighting** | **Data Source** |
| --- | --- | --- | --- | --- |
| Landcover | Evergreen forest, mixed forest, forest | Reclassify | 15% | Global Forest Watch^1^ |
| Rivers | Habitat suitability increasing with proximity to water | Negative Linear | 20% | FAO^[[2]](#footnote-2)^ |
| Roads | Prefer sparse density | Linear | 8% | Open Street Map^[[3]](#footnote-3)^ |
| Settlements | Prefer greater distance | Linear | 22% | Open Street Map |
| Deforestation Hot Spots | Avoidance of anthropogenic disturbance | Linear | Historic (2001 – 2015): 25% | Global Forest Watch2 |
|  |  |  | Recent (2001 – 2020): 10% |  |

1. Hansen, M. C., P. V. Potapov, R. Moore, M. Hancher, S. A. Turubanova, A. Tyukavina, D. Thau, S. V. Stehman, S. J. Goetz, T. R. Loveland, A. Kommareddy, A. Egorov, L. Chini, C. O. Justice, and J. R. G. Townshend. 2013. “High-Resolution Global Maps of 21st-Century Forest Cover Change.” Science 342 (15 November): 850–53. Data available from: https://glad.umd.edu/dataset/global-2010-tree-cover-30-m. [↑](#footnote-ref-1)
2. Map data copyrighted OpenStreetMap contributors and available from https://www.openstreetmap.org [↑](#footnote-ref-2)
3. River networks of Cambodia - OD Mekong Datahub. (2022). Accessed: 2 August 2022. https://data.opendevelopmentcambodia.net//dataset/river-networks-of-cambodia [↑](#footnote-ref-3)
