# Supplementary material for: Giants in the landscape: status, genetic diversity, habitat suitability and conservation implications for a fragmented Asian elephant (Elephas maximus) population in Cambodia
Source: PeerJ. 2025 Mar 13;13:e18932. doi: 10.7717/peerj.18932 (PMC11910960; doi:10.7717/peerj.18932)
Supplement: Supplemental Information 8 [file peerj-13-18932-s008.docx]

**Table S4**

**Resistance values for variables included within functional connectivity models**

| **Variable** | **Resistance Value** |
| --- | --- |
| Forest^[[1]](#footnote-1)^^[[2]](#footnote-2)^^[[3]](#footnote-3)^ | 5 |
| Non-forest | 100 |
| Settlements^[[4]](#footnote-4)^ | 100 |
| Roads^[[5]](#footnote-5)^^[[6]](#footnote-6)^ | 75 |

1. Liu, S., Yin, Y., Li, J., Cheng, F., Dong, S., Zhang, Y., 2018. Using cross-scale landscape connectivity indices to identify key habitat resource patches for Asian elephants in Xishuangbanna, China. Landscape and Urban Planning 171, 80–87. [↑](#footnote-ref-1)
2. Huang, C., Li, X., Khanal, L., Jiang, X., 2019. Habitat suitability and connectivity inform a co-management policy of protected area network for Asian elephants in China. PeerJ 7, e6791. https://doi.org/10.7717/peerj.6791 [↑](#footnote-ref-2)
3. Puyravaud, J.-P., Cushman, S.A., Davidar, P., Madappa, D., 2016. Predicting landscape connectivity for the Asian elephant in its largest remaining subpopulation. Anim Conserv 20, 225–234. https://doi.org/10.1111/acv.12314 [↑](#footnote-ref-3)
4. Torre, J.A., Lechner, A.M., Wong, E.P., Magintan, D., Saaban, S., Campos‐Arceiz, A., 2019. Using elephant movements to assess landscape connectivity under Peninsular Malaysia’s central forest spine land use policy. Conservat Sci and Prac 1. https://doi.org/10.1111/csp2.133 [↑](#footnote-ref-4)
5. Suksavate, W., Duengkae, P., Chaiyes, A., 2019. Quantifying landscape connectivity for wild Asian elephant populations among fragmented habitats in Thailand. Global Ecology and Conservation 19, e00685. https://doi.org/10.1016/j.gecco.2019.e00685 [↑](#footnote-ref-5)
6. Wadey, J., Beyer, H.L., Saaban, S., Othman, N., Leimgruber, P., Campos-Arceiz, A., 2018. Why did the elephant cross the road? The complex response of wild elephants to a major road in Peninsular Malaysia. Biological Conservation 218, 91–98. https://doi.org/10.1016/j.biocon.2017.11.036 [↑](#footnote-ref-6)
