# Supplementary material for: Giants in the landscape: status, genetic diversity, habitat suitability and conservation implications for a fragmented Asian elephant (Elephas maximus) population in Cambodia
Source: PeerJ. 2025 Mar 13;13:e18932. doi: 10.7717/peerj.18932 (PMC11910960; doi:10.7717/peerj.18932)
Supplement: Supplemental Information 9 [file peerj-13-18932-s009.docx]

**Supplementary Table S5.**

**Summary of the 35 unique genotypes identified in the sample set.**

| Unique genotype ID | Mitochondrial haplotype | Sex | Sample count | Location |
| --- | --- | --- | --- | --- |
| UG01 | AC (or III) | Male | 8 | Prey Lang |
| UG02 | AC (or III) | Male | 3 | Prey Lang |
| UG03 | AC (or III) | Male | 11 | Prey Lang |
| UG04 | Unknown | Male | 1 | Prey Lang |
| UG05 | AAAK (or II) | Male | 3 | Prey Lang |
| UG06 | AAAK (or II) | Female | 6 | Prey Lang |
| UG07 | AC (or III) | Male | 2 | Prey Lang |
| UG08 | AC (or III) | Male | 1 | Prey Lang |
| UG09 | AC (or III) | Male | 2 | Prey Lang |
| UG10 | AC (or III) | Female | 2 | Prey Lang |
| UG11 | AC (or III) | unknown | 1 | Prey Lang |
| UG12 | AC (or III) | Male | 2 | Prey Lang |
| UG13 | AC (or III) | Male | 1 | Prey Lang |
| UG14 | AC (or III) | Female | 2 | Prey Lang |
| UG15 | Unknown | Female | 1 | Prey Lang |
| UG16 | AC (or III) | Male | 1 | Prey Lang |
| UG17 | ADAE (or IV) | Female | 4 | Prey Lang |
| UG18 | ADAE (or IV) | Female | 3 | Prey Lang |
| UG19 | ADAE (or IV) | Female | 1 | Prey Lang |
| UG20 | ADAE (or IV) | Male | 2 | Prey Lang |
| UG21 | ADAE (or IV) | Female | 1 | Prey Lang |
| UG22 | ADAE (or IV) | Male | 1 | Prey Lang |
| UG23 | BN (or I) | Female | 8 | Preah Roka/Chhaeb |
| UG24 | BN (or I) | Male | 8 | Preah Roka/Chhaeb |
| UG25 | BN (or I) | Male | 5 | Chhaeb |
| UG26 | BN (or I) | Female | 7 | Preah Roka/Chhaeb |
| UG27 | ADAE (or IV) | Female | 1 | Preah Roka |
| UG28 | ADAE (or IV) | Female | 7 | Preah Roka/Chhaeb |
| UG29 | ADAE (or IV) | Female | 4 | Preah Roka/Chhaeb |
| UG30 | Unknown | Male | 8 | Preah Roka/Chhaeb |
| UG31 | ADAE (or IV) | unknown | 1 | Preah Roka |
| UG32 | Unknown | unknown | 1 | Chhaeb |
| UG33 | Unknown | Male | 1 | Chhaeb |
| UG34 | Unknown | Female | 1 | Chhaeb |
| UG35 | Unknown | Female | 1 | Chhaeb |
