# Supplementary material for: Giants in the landscape: status, genetic diversity, habitat suitability and conservation implications for a fragmented Asian elephant (Elephas maximus) population in Cambodia
Source: PeerJ. 2025 Mar 13;13:e18932. doi: 10.7717/peerj.18932 (PMC11910960; doi:10.7717/peerj.18932)
Supplement: Supplemental Information 10 [file peerj-13-18932-s010.docx]

**Supplementary Article S1 - Genetic study methods**

*Swab sample collection*

The swab was first soaked in DNA stabilization buffer that was in pre-filled tubes (750ul per 2ml tube) and then used to swab the entire surface of the bolus making sure to target areas with glistening mucus first. The swab was re-soaked in the buffer during this process and twirled to release cells into the tube. Once the entire surface had been sampled the swab was placed in the tube, the handle snapped off and the screw-lid tightened.

*Ethanol sample collection*

A 50ml tube was prefilled with 25ml of absolute ethanol. External chunks of the dung bolus were extracted with a spoon and placed into the tube containing ethanol until the ethanol reached the 50ml level. The screw cap was then tightened and the seal between the cap and tube was sealed using parafilm wrap. All tubes were labelled with a unique identification number and a sample using the swab method and then the ethanol method was collected for every dung pile. If too many samples were encountered, then sampling focused on the freshest samples as determined by the system outlined in Hedges (2012).

*DNA extraction*

Although DNA extraction from both sample types used the QIAamp® Fast DNA Stool Mini kits there were some slight differences in the initial steps.

For the Swab samples, before starting the extraction all samples were checked that the swab was submerged within the buccal fix remaining in the tube. If the swab was not fully submerged, additional buccalfix was added to the tube. All samples were then left for at least 30 minutes submerged in the solution before beginning the extraction. Then using disinfected tweezers, the swab head was turned upside down, with the swab head at the top of the tube. It was then centrifuged at 4000rpm for 1minute. The swab was then removed and discarded. InhibitEx Buffer (500μl) was then added to the tube and pulse vortexed for ~15seconds. The tube was then centrifuged for 1 minute at 14,000 rpm and 600 µl of the supernatant was added to a 2ml flip-top tube containing 25µl of proteinase K. The methods then followed the manual with two elutions created after the final wash stages, the first using 75µl of ATE and the second using 50µl.

For the ethanol samples, all samples were checked to ascertain whether the dung sample was still submerged in ethanol. A few samples required additional ethanol adding to fill them up to the 50ml line, if this was conducted the sample was shaken vigorously and then left at room temperature overnight before starting extraction. When beginning the extraction, the sample was mixed well by vigorously shaking, then 1500µl of sample was pipette into a 2ml tube. If the sample contained a lot of faecal matter, the end of the pipette tip was cut off to make it easier, and for samples with an excess of solid matter, only 500µl of sample was pipette into the tube. The sample was then centrifuged for 5mins at 14,000rpm or until all the solid matter was pelleted to the bottom of the tube. All of the supernatant was then pipette off and discarded. The sample was then centrifuged again for 1min at 14,000rpm and any further supernatant dicarded. The tubes were then placed on a thermoblock set at 35°C for 30mins or until the sample was dry. Then 1ml of InhibitEx buffer was added to the tube and the sample vortexed for 2 minutes or until the sample was distributed throughout the buffer. The sample was then left to stand for up to 30 minutes at room temperature before it was centrifuged for 1 minute at 14,000rpm. Then 600 µl of the supernatant was added to a 2ml flip-top tube containing 25µl of proteinase K. The extraction then followed the manual with two elutions created after the final wash stages, the first using 75µl of ATE and the second using 50µl.

*SNP genotyping*

The genotyping using the 20 SNPs was conducted using KASP by-design assays run on a StepOne™ Real-Time PCR System (Thermo Fisher Scientific, Waltham, MA, USA). All DNA extracts were diluted 1:5 before use and genotyping conducted in 48-well plates. Every plate contained at least 3 positive controls (including a homozygote 1, a homozygote 2 and a heterozygote control) and 2 negative controls. The master mix was created so that each well contained 5μl High-ROX KASP-BIO master mix and 0.14μl of the KASP assay. A total of 3μl of template DNA was used in each reaction. The PCR thermocycling protocol was as follows:

1. 94°C for 15 min

2. 94°C for 20 sec

3. 65°C for 1 min - Decrease by 1°C every cycle

4. Cycle to step 2 for 8 more cycles

5. 94°C for 20 Sec

6. 57°C for 1 min

7. Cycle to step 5 for 35 more cycles

8. 21°C and conduct a fluorescent read

*Microsatellite genotyping*

The microsatellite genotyping was conducted in two multiplex panels, one containing 5 markers and the other 4, as identified in Table S2 via P1 and P2. DNA extracts were diluted 1:5. All primers were diluted to 20mM before creating primer mixes of approximately 100μl volumes. The same volume of forward primer was added as its complimentary reverse primer, however, the ratios of each marker differed in the primer mixes as outlined in the tables below:

| **Primer Mix 1 (P1)** | | |
| --- | --- | --- |
| **Primer** | **Dye** | **Strength** |
| Emu03 | NED | 4x |
| Emu04 | FAM | 3x |
| Emu07 | VIC | 1.5x |
| Emu12 | PET | 12x |
| FH48 | FAM | 3x |

| **Primer Mix 2 (P2)** | | |
| --- | --- | --- |
| **Primer** | **Dye** | **Strength** |
| Emu10 | FAM | 2x |
| Emu15 | NED | 3x |
| Emu17 | VIC | 1.5x |
| LafMS03 | PET | 11x |

All PCR reactions were conducted in 10μl volumes. A master mix was created so that each reaction contained 5µl Qiagen Type-It, 1µl Qiagen Q-Solution, 2µl ddH2O and 1µl Primer mix. A total of 1µl of template DNA was used in each reaction. Both multiplexes used the same thermocycling protocol as outlined below:

1. 95˚C for 5 min
2. 95˚C for 40 sec
3. 56˚C for 55 sec
4. 72˚C for 40 sec
5. Cycle to step 2 for 39 more cycles
6. 60˚C for 30 min

PCR products were then run on an Applied Biosystems 3730XL DNA analyser and genotyping conducted in Geneious Prime v 2021.1.1

*Mitochondrial sequencing*

Two overlapping fragments of the d-loop mitochondrial region were amplified via PCR. The primers used in the PCR for each fragment were designed using Primer3 (Untergasser *et al.* 2012) and can be found in Table S2. All primers were diluted to 10µM before use and PCR reactions were conducted in 20µl volumes. Each reaction contained 10µl DreamTaq Hot Start, 2µl Forward primer [10µM], 2µl Reverse primer [10µM] and 4.5µl nuclease Free water. The PCR thermocycle profile for fragment 1 (AEL_dloop) was as follows:

1. 95°C for 5min
2. 95°C for 30sec
3. 55°C for 30sec
4. 72°C for 60sec
5. Cycle to step 2 for 29 more cycles
6. 72°C for 10min

Due to problems with multi-banding a touchdown PCR protocol was used for fragment 2 (CR_AEL_RZSS) as follows:

1. 95°C for 5min
2. 95°C for 20sec
3. 60°C for 30sec
4. 72°C for 30sec
5. Cycle to step 2 for 4 more cycles
6. 95°C for 20sec
7. 58°C for 30sec
8. 72°C for 30sec
9. Cycle to step 6 for 4 more cycles
10. 95°C for 20sec
11. 56°C for 30sec
12. 72°C for 30sec
13. Cycle to step 10 for 4 more cycles
14. 95°C for 20sec
15. 54°C for 30s
16. 72°C for 30s
17. Cycle to step 14 for 29 more cycles
18. 72°C for 7min
19. 4°C for 20min

PCR products were visualised on a 1.5% agarose gel, prepared using BigDye reactions and sequenced on an Applied Biosystems 3730 DNA analyser. The sequences from the two fragments and then aligned to create a consensus for each sample in Geneious Prime v 2021.1.1. This led to the creation of a final 152bp sequence for each successful sample.

*References*

Hedges, S. 2012. Estimating elephant population density and abundance from dung pile density: theoretical concepts. – In: Hedges, S.(ed.), Monitoring elephant populations and assessing threats. A manual for researchers, managers and conservationists. Universities Press (India) Private Limited, pp. 61–111.

Untergasser A, Cutcutache I, Koressaar T, Ye J, Faircloth BC, Remm M, Rozen SG. Primer3--new capabilities and interfaces. Nucleic Acids Res. 2012 Aug;40(15):e115. doi: 10.1093/nar/gks596. Epub 2012 Jun 22. PMID: 22730293; PMCID: PMC3424584.
